# A Case of Severe Thrombocytopenia, Aseptic Meningitis, and Hepatitis Caused by Trimethoprim-Sulfamethoxazole: A Triple Threat

**DOI:** 10.7759/cureus.65945

**Published:** 2024-08-01

**Authors:** Srikanth R Kothapalli, Meghana Kesireddy

**Affiliations:** 1 Internal Medicine, CHI Health St. Francis, Grand Island, USA; 2 Hematology-Oncology, The University of Nebraska Medical Center, Omaha, USA

**Keywords:** hepatitis, aseptic meningitis, thrombocytopenia, idiosyncratic reactions, drug-induced adverse events, trimethoprim-sulfamethoxazole

## Abstract

Trimethoprim-sulfamethoxazole (TMP-SMX), a widely used antibiotic, is associated with both predictable dose-dependent side effects and rare, idiosyncratic adverse reactions. Here, we report the case of a previously healthy, non-G6PD-deficient, 27-year-old male who developed three idiosyncratic reactions: severe thrombocytopenia, aseptic meningitis, and hepatitis concurrently following TMP-SMX administration. The Naranjo adverse reaction probability score was 7, implying TMP-SMX as the probable cause of the clinical presentation. After a comprehensive workup to rule out alternate etiologies, we have established TMP-SMX as the culprit. Our case highlights the importance of early recognition of TMP-SMX-induced rare adverse events for appropriate management to mitigate long-term sequelae and ensure favorable patient outcomes.

## Introduction

Trimethoprim-sulfamethoxazole (TMP-SMX) is a commonly used antibiotic to treat various bacterial infections and as prophylaxis and treatment for pneumocystis carinii pneumonia and toxoplasmosis. SMX, a sulfonamide, inhibits dihydrofolic acid formation from para-aminobenzoic acid, blocking bacterial folic acid synthesis. TMP inhibits dihydrofolate reductase, preventing the reduction of dihydrofolic acid to tetrahydrofolate, which is essential for purine synthesis needed for DNA and protein production. This combination of TMP and SMX creates a synergistic anti-folate effect [1]. TMP-SMX can cause pharmacodynamically predictable, dose-dependent side effects (type A reactions) like mild gastrointestinal symptoms, bone marrow suppression, megaloblastic changes, hyperkalemia, etc. It can also cause rare, idiosyncratic, dose-independent side effects (type B reactions) such as liver failure, skin rashes like exfoliative dermatitis, Stevens-Johnson syndrome, sepsis-like hypersensitivity reactions, drug-induced immune thrombocytopenia, etc. [2]. Here, we report a case of a young, healthy man without glucose 6-phosphate dehydrogenase (G6PD) deficiency who developed three concurrent idiosyncratic reactions: severe thrombocytopenia, aseptic meningitis, and hepatitis due to TMP-SMX.

## Case presentation

**Figure 1:**
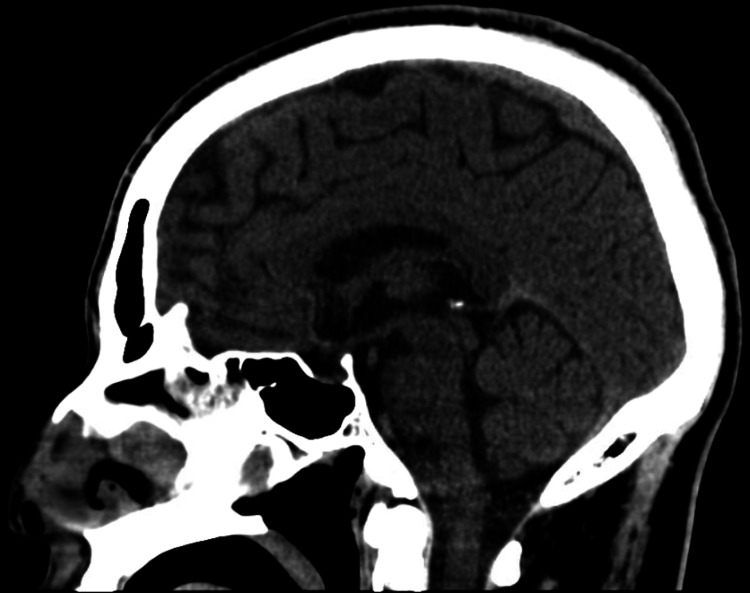
CT head showing normal findings

A 27-year-old male with no significant past medical history presented with a 3-day history of fever, headaches, neck stiffness, and a rash on his extremities. Seven days before his symptoms began, he was started on TMP-SMX twice daily for 14 days for a possible urinary tract infection. This was the first time he was given TMP-SMX, and he was not taking any other medications or over-the-counter drugs during this period. The examination was significant for a temperature of 39.2 °C, a heart rate of 110/min, neck stiffness, and a diffuse petechial rash of the bilateral upper and lower extremities. Labs on admission showed a platelet count of 0 cells/mcL, normal hemoglobin (13.5 g/dl) and white blood cell count (4.2 cells/mcL), elevated alanine aminotransferase/ALT 86 U/L (normal 7-52 U/L), elevated aspartate aminotransferase/AST 74 U/L (normal 15-41 U/L), normal alkaline phosphatase (70 U/L), and total bilirubin (0.6 mg/dl). A computed tomography (CT) scan of the head was normal without any acute bleeding (Figure 1). A peripheral smear confirmed marked thrombocytopenia with morphologically normal white blood cells and no blasts. An extensive workup, including tests for infectious, hemolytic, and auto-immune causes, is summarized in Table 1 and was all unrevealing.

**Table 1:** Laboratory work-up Ig: immunoglobulin; Ab: antibody

Test	Result	Reference range
Hemolysis		
Haptoglobin	188 mg/dl	36–215 mg/dl
Glucose 6-phosphate dehydrogenase	11.7 U/g Hgb	9.9–16.6 U/g Hb
Infections		
Erythrocyte sedimentation rate (ESR)	6 mm/hr	0–15 mm/hr
C-reactive protein (CRP)	0.9 mg/dl	<1 mg/dl
Blood cultures X 2	No growth	
Urinalysis	Mild hematuria, no evidence of urinary tract infection	
COVID-19 PCR	Negative	
Human immunodeficiency virus (HIV)	HIV Ag-Ab: non-reactive, HIV 1 p24 Antigen: non-reactive, HIV 1 Ab: non-reactive, HIV 2 Ab: non-reactive	
Hepatitis B virus	Hep B surface antigen: non-reactive, Hep B surface antibody: non-reactive, Hep B core antibody: non-reactive	
Hepatitis C virus	Hep C antibody: non-reactive	
Epstein-Barr virus (EBV)	EBV VCA IgM: negative, EBV VCA IgG: positive, EBV EA(D) IgG: negative, EBNA IgG: positive → Indicates past infection	
Cytomegalovirus	IgM Ab: negative, IgG Ab: positive → Indicates past infection	
Lyme disease	Antibody: 0.74 LIV: negative	0.99 LIV or less: negative
West Nile virus	IgM Ab: negative, IgG Ab: positive → Indicates past infection	
Rocky Mountain spotted fever (Rickettsia rickettsii antibody)	IgM Ab: <1:64: negative, IgG Ab: <1:64: negative	<1:64: negative
Mumps antibody	IgM Ab 0.26 IV: negative, IgG Ab: reactive → Indicates past infection or immunity	0.79 IV or less: negative
Ehrlichia	IgM Ab <1:16: negative, IgG Ab <1: 64: negative	IgM <1:16: negative, IgG <1:64: negative
Tick-borne disease panel by polymerase chain reaction (PCR) on blood: Babesia species, *Babesia microti*, *Anaplasma phagocytophilum*, *Ehrilichia chaffeensis*, *Ehrilichia ewingii*/*canis*, *Ehrilichia muris*-like	Not detected	
Blood smear for parasites like Babesia, Anaplasma	Negative for parasites	
Other		
Anti-nuclear antibody titer	<1:40: negative	<1:40: negative
Coagulation labs		
PT	13.7 sec	10.1–14.2 sec
INR	1.1	0.9–1.1
PTT	35 sec	24–36 sec
Fibrinogen	206 mg/dl	160–450 mg/dl

The Naranjo adverse reaction probability score was 7, indicating a probable drug reaction to TMP-SMX as the cause of his clinical presentation (Table 2) [3]. The World Health Organization - Uppsala Monitoring Centre (WHO-UMC) causality assessment score also indicated TMP-SMX as the probable or likely cause [4].

**Table 2:** Naranjo adverse drug reaction probability scale

Question	Yes	No	Do not know	Score
1. Are there previous conclusive reports on this reaction?	+1	0	0	+1
2. Did the adverse event appear after the suspected drug was administered?	+2	−1	0	+2
3. Did the adverse event improve when the drug was discontinued or a specific antagonist was administered?	+1	0	0	+1
4. Did the adverse event reappear when the drug was readministered?	+2	−1	0	0
5. Are there alternative causes that could on their own have caused the reaction?	−1	+2	0	+2
6. Did the reaction reappear when a placebo was given?	−1	+1	0	0
7. Was the drug detected in blood or other fluids in concentrations known to be toxic?	+1	0	0	0
8. Was the reaction more severe when the dose was increased or less severe when the dose was decreased?	+1	0	0	0
9. Did the patient have a similar reaction to the same or similar drugs in any previous exposure?	+1	0	0	0
10. Was the adverse event confirmed by any objective evidence?	+1	0	0	+1
	Total Score: 7			

The TMP-SMX was discontinued immediately upon admission. With no other causes identified for thrombocytopenia, such as disseminated intravascular coagulation ruled out with normal coagulation labs, thrombocytopenic purpura/hemolytic uremic syndrome ruled out with negative hemolysis, heparin-induced thrombocytopenia ruled out due to lack of heparin exposure, negative tests for human immunodeficiency virus (HIV), hepatitis B/C, Epstein-Barr virus, cytomegalovirus, and no evidence of auto-immune disease or other drug exposure, these findings supported a diagnosis of TMP-SMX-induced thrombocytopenia. Due to severe thrombocytopenia with microscopic hematuria and petechiae, he was started on prednisone 1 mg/kg immediately on admission and intravenous immunoglobulin (IVIG) at a dose of 1 g/kg for two doses on days 2 and 3. This led to rapid platelet recovery (trend shown in Table 2), and steroids were tapered over a total of six weeks.

**Table 3:** Platelet count, ALT, and AST trend ALT: alanine aminotransferase, AST: aspartate aminotransferase

	Day 1	Day 2	Day 3	Day 4	Day 5	2 weeks after admission	2 years after admission
Platelet count (cells/mcL)	0 (started prednisone 1mg/kg)	1,000 (1^st^ dose of IVIG 1g/kg)	1,000 (2^nd^ dose of IVIG 1g/kg)	16,000	73,000	260,000	232,000
ALT (normal: 7-52 U/L)	86	116	119	102	83	25	14
AST (normal: 15-41 U/L)	74	72	59	62	35	14	18

For suspected meningitis (fever, headache, neck stiffness), a lumbar puncture was not performed due to severe thrombocytopenia. Empiric antibiotics, Vancomycin and Ceftriaxone, were started for possible bacterial meningitis, especially given a fever of 39.2 °C on presentation, but were discontinued after 48 hours of negative blood cultures and no additional fevers. The complete resolution of headaches and neck stiffness within a day of stopping TMP-SMX, along with the extensive negative infectious workup and no other alternate etiologies, supported a diagnosis of TMP-SMX-induced aseptic meningitis. The hepatitis spontaneously resolved (trend shown in Table 2), with no alternative etiologies like ischemia, hepatitis B/C, or other drug exposure, supporting the diagnosis of TMP-SMX-induced hepatitis. He was discharged on day 5 of hospitalization. He had no recurrences for over two years since the initial presentation, with no further exposure to TMP-SMX.

## Discussion

Rare drug-induced adverse events are often reported through case reports/studies and adverse event reporting databases. However, these reports may be influenced by various factors, making it challenging to determine the precise association between drug exposure and adverse reactions. The Naranjo adverse drug reaction probability scale, consisting of 10 questions, is widely used to establish causality [3]. It is worth noting that some questions in the Naranjo probability scale, such as the event reappearance upon drug readministration or detection of the drug in toxic levels in the blood or body fluids, are impractical or unethical to assess, making it difficult to establish the drug as the definitive cause of the adverse event. Our patient had a score of 7 on the Naranjo scale for all three reactions - thrombocytopenia, aseptic meningitis, and hepatitis - making TMP-SMX the probable cause of his clinical presentation. The World Health Organisation-Uppsala Monitoring Centre (WHO-UMC) causality assessment score also indicated TMP-SMX as the probable or likely cause [4]. Another criterion proposed by George et al. for evaluating drug-induced thrombocytopenia is simpler, with only four criteria [5]. Our patient met criteria 1-3, providing level II evidence suggesting TMP-SMX as a probable cause. Achieving level I evidence (definite cause) on George et al. criteria requires meeting criterion 4, which is the development of recurrent thrombocytopenia upon re-exposure to the drug. However, it is unethical to re-challenge solely for documenting level I evidence, given the severity of thrombocytopenia in our patient on presentation and also because the adverse event can sometimes worsen with re-exposure.

Drugs can cause thrombocytopenia either by a direct toxic effect on the bone marrow or through immune-mediated peripheral destruction of platelets [6,7]. The incidence of drug-induced thrombocytopenia in the general population is estimated at only 10 to 18 cases per 1 million population per year [6,8]. Sulfonamides, like TMP-SMX, are frequently implicated. Despite this frequent association, the risk of developing acute thrombocytopenia (platelet count ≤30,000/mcL) along with petechiae, bruising, or bleeding was relatively low, with a risk estimate of 38 cases per million users in a week [8]. The mechanism of TMP-SMX-induced thrombocytopenia is not fully understood, but it is thought to be due to the development of drug-dependent antibodies against the platelet membrane glycoproteins (GP) Ib/IX and GP IIb/IIIa [6]. The onset of thrombocytopenia typically occurs after four days to five weeks of drug exposure [9]. Treating TMP-SMX-induced thrombocytopenia involves stopping TMP-SMX, which is often sufficient in most cases for complete platelet count recovery. In severe cases, systemic corticosteroids or IVIG may be necessary. Systemic corticosteroids work through their immune-suppressive and thrombopoietic effects, are effective in 90% of cases, and can be tapered over four to six weeks. IVIG, at a dose of 1 g/kg daily for 2 days or 0.4 g/kg daily for five days, rapidly increases platelet count by saturating platelet receptors and preventing the binding of drug-induced antiplatelet antibodies. However, IVIG is expensive and is typically used when corticosteroids fail or are not feasible in cases of uncontrolled diabetes or infections, or when a rapid increase in platelet count is necessary [10,11]. Platelet transfusions may be used in cases of active bleeding or to prevent major bleeding when platelet counts are extremely low (<10,000 cells/mcl); however, their efficacy may be limited if drug-induced platelet destruction persists.

Drug-induced aseptic meningitis is rare, with nonsteroidal anti-inflammatory drugs (NSAIDs) being the most commonly associated drugs. TMP-SMX is one of the anti-microbial drugs most linked to aseptic meningitis [12]. The exact pathogenesis of TMP-SMX-induced aseptic meningitis is unclear but is thought to be either a type II or III hypersensitivity reaction triggered by hapten conjugation with a cerebrospinal fluid (CSF) or meningeal protein or a type IV hypersensitivity reaction triggered by T-cell activation [13]. The time to onset of symptoms ranges from a few hours to three months of drug exposure, and symptoms can reappear within 12 hours of re-exposure to TMP-SMX. Common symptoms include fever, headaches, altered mental status, nausea and vomiting, and neck pain. CSF analysis usually shows elevated white blood cells and protein, normal glucose, and a negative infectious workup. Symptoms generally resolve rapidly with full recovery, typically within 48 to 72 hours, but relapse upon re-administration of the drug [14].

Drug-induced hepatotoxicity is most commonly associated with acetaminophen [15]. TMP-SMX infrequently causes liver injury, with severity ranging from a mild, transient increase in serum aminotransferases to rare cases of fulminant hepatic failure [16]. A retrospective study on TMP-SMX for pneumocystis carinii pneumonia prophylaxis found thrombocytopenia (37%) and liver dysfunction (20%) as the most common reasons for discontinuation, though this may have been confounded by the underlying disease and concomitant medications [17]. The latency to the onset of hepatitis varies from 2 to 12 weeks after initial ingestion [18]. The mechanism of TMP-SMX-induced hepatitis is not fully understood but is thought to involve either idiosyncratic reactions or reactive metabolites causing direct hepatocyte damage. Our patient had a mild hepatocellular injury pattern (with elevated AST and ALT; normal Alk phosphatase and bilirubin), but TMP-SMX can also cause cholestatic or mixed-pattern hepatic injury [18]. Hepatitis typically spontaneously resolves on drug cessation, but in rare cases, it can progress to acute liver failure, hepatic necrosis, or death [19].

TMP-SMX-induced drug hypersensitivity syndromes commonly affect the skin (maculopapular rash) and liver and are associated with fever and aseptic meningitis. Susceptibility to drug hypersensitivity syndromes depends on genetic and immune factors, with immunocompromised patients and those with G6PD deficiency being more vulnerable to TMP-SMX-induced drug hypersensitivity syndromes and toxicity [20]. However, our patient, who is not immunocompromised and does not have G6PD deficiency, developed the three idiosyncratic reactions concurrently along with fever, which is the first reported to our knowledge.

## Conclusions

This case underscores the importance of recognizing TMP-SMX-induced rare adverse events, especially when they occur together and lack an alternative unifying diagnosis to explain the clinical presentation. Recognition of these rare but potentially life-threatening adverse events can prevent excess workup, prompt withdrawal of the drug, complete recovery without any long-term sequelae, and unintended re-exposure to the drug. Our report, the first to document three simultaneous idiosyncratic reactions in a young, healthy, non-G6PD-deficient person, adds to the existing literature and enhances physician awareness.
